# Migrations, arts, and bodies: the Silhouette in multiple shadows of Rubiane Maia

**DOI:** 10.3389/fsoc.2025.1694064

**Published:** 2025-12-22

**Authors:** Paula Guerra

**Affiliations:** Department of Sociology, Faculty of Arts and Humanities, Institute of Sociology of University of Porto, Griffith Centre for Social and Cultural Research, Porto, Portugal

**Keywords:** migration, contemporary art, borders, identities, body

## Abstract

This article explores the work of the Brazilian artist Rubiane Maia, currently based in the United Kingdom, focusing on two of her performances as a privileged sociological field for analyzing migration, corporeality, and contemporary art. The main objective of this article is to examine how the artist mobilizes interdisciplinary practices to interrogate memories, displacements, and relations between humans and more-than-humans, focusing on two artistic creations: the *Book-Performance* and the *Speirein*. Through a qualitative approach, the analysis highlights how Maia choreographs border experiences, challenging fixed identities and proposing new ways of inhabiting both body and territory. Our study demonstrates how the artist transforms the vulnerability of the migrant body into a creative force, establishing critical dialogues with collective memory and the public sphere.

## 1 Rubiane’s urgency to choreograph the (im)possible^1^

*The infinite possibilities that open up before the desire (and urgency) to choreograph the impossible*. (Maia, 2023b, n.p.)

This quote illustrates how Rubiane Maia articulates her relationship with art, the world, and its limitless dialogical possibilities. The artist,^2^ born in 1984 in Espírito Santo, Brazil and now based in the United Kingdom, has developed an experimental body of work that spans performance, installation, and other artistic languages such as writing, photography, video, and painting. Her practice sharpens the audience’s senses to the potential synergy between human and more-than-human relations—minerals, plants, soils. A notable example is *In the Time of Flying Fishes* (2022), the result of an artistic residency in the Canary Islands (Maia, 2023b). In this project, Maia elaborates a symbolic matrix with an archaeological foundation, treating the earth as a dermis saturated with secrets from which she reflects upon memories and traces of annihilated existences.^3^ In her own words:

*I think performance was my first urgency, but as I began to perform, I realized that it opens many possibilities. I started to understand myself as an artist-researcher, working with conceptual elements. I like to engage in research processes that move into practice, into bodily experimentation, but also along a theoretical path that runs through language. I also arrived at this place … of moving across media. Now I am working on a project that is moving towards painting with earth; I started working with earth in performance, but now the earth is assuming other roles—as a living matter I can use differently and engage with through new processes. (Rubiane Maia, interview, April 2024)*

Grounded in autobiographical concerns and in contexts of research, immersion, and artistic residencies, Maia entered the “in-between” zone that marked her childhood and youth (Anzaldúa, 1987; Butler, 1997a; Lugones, 1992). Today, among the aspects that most profoundly shape her work are the complexities of separation and belonging. This perspective is consonant with her place of speech [*lugar de fala*] (Ribeiro, 2017), the solid foundations of which are inscribed in her Black diasporic body, itself the product of colonial historical processes that long preceded her birth (Bönisch-Brednich et al., 2023). Accordingly, this article explores crucial dimensions of Maia’s oeuvre, including the body, raciality, migration, and lived subjectivity within the Brazilian and international performance art fields (Schechner, 2017; Schneider, 2013). It is framed as a case study that illuminates the ongoing challenges of being a woman, Black, a mother, born in the Global South, and a migrant (Butler, 2004). This inquiry is situated within the dialectical, multidisciplinary sphere of the sociology of the arts and its intersections with the sociology of migration, the sociology of the body, art history, feminist studies, cultural studies, and post-subcultural studies (Guerra, 2024a, 2025b).

Indeed, this article lies at the core of my research trajectory, which has consistently been shaped by a commitment to analyzing the lived experiences and creative productions of women artists in their plurality, as well as to exploring emergent artistic fields. In Portugal, where we initially focused on women artists’ trajectories, we were able to identify both regularities and socio-historical singularities (Guerra, 2020a,b, 2021a). When confronted with realities from other regions, especially Brazil, these findings revealed new dimensions and challenges for sociological research (de Boise, 2016). In the Portuguese context, my work underscored the construction of artistic identities embedded within a rich historical-cultural tradition that, while significant, presented limitations in representing the plurality of female experiences. The search for more complex narratives of inequality ultimately led us to turn towards Brazil. With its intricate heritage, Brazil offers fertile ground to reconfigure notions of art and gender, as well as agency and structure, through an intersectional lens (Guerra, 2021b, 2023a,b; Guerra and Oliveira, 2023; de Oliveira and Guerra, 2021a,b). In examining Brazilian women artists, I encountered trajectories that, while sharing points of convergence with some Portuguese counterparts, also exhibit specificities that challenge established paradigms.

It was in this context that I encountered Rubiane Maia. In this article, we focus on two of her performances, *Book-Performance* and *Speirein*, which engage directly with migratory processes—particularly traumatic ones—as articulated by the artist-researcher herself. Traumatic migration here is conceived as a process of dislocation that entails the fragmentation and destruction of identity bonds, with expanded consequences for the migrant body and subjectivity (Theisen-Womersley, 2021; Vlase, 2024). Maia’s works speak directly to this perspective: her performance practice functions as a sensitive testimony to the invisible scars left by such processes, mobilizing the body as a living archive capable of exposing these political and affective marks (Bönisch-Brednich et al., 2023; Kumar and Triandafyllidou, 2023).

This study is grounded in a life-history approach to illuminate gender inequalities, migratory processes, exclusion, and strategies of contestation from the vantage point of artistic creation, specifically within art-life processes, as we have previously argued (Guerra, 2024a, 2024b, 2025a; Taylor, 2025). The life-history method, allows for a situated analysis of Rubiane Maia’s artistic and migratory trajectory. In the present study, this perspective proves particularly appropriate, since Rubiane Maia’s artistic and migratory journey unfolds through successive biographical ruptures that intertwine body, identity, and creation.

The article interrogates the factors that perpetuate women’s invisibility in the arts, particularly within migration contexts, while examining how the artist employs her practice to engage politically with the public sphere, foregrounding fractures and structural inequalities inscribed and reproduced in her body and art—always “in between.” Methodologically, the approach is qualitative. It is worth underscoring that qualitative inquiry consolidated its place in the sociology of the arts through post-positivist critiques and the affirmation of interpretive, constructivist, and critical paradigms (DeNora, 2000; Denzin and Lincoln, 2023; Hennion, 1993), resonating^4^ with Eco’s (1971) notion of the open work. By privileging processes, meanings, and lived experiences, this methodology emphasizes the social production of meaning—a particularly relevant lens through which to study Maia’s trajectory and artistic practice (Alves, 2021; Danto, 1989; Fourmentraux, 2005). Accordingly, I developed a categorical content analysis applied both to Maia’s narrative—particularly a semi-structured interview—and to her artistic creations, outlined above. This analysis centered on three analytical dimensions: migratory process, lived experience, and artistic creation. Following these dimensions, the article is structured around three dialogical movements between theory and empirics: first, the migratory trajectory of Rubiane; second, the thematic of the body; and third, the feminist epistemologies embodied in Rubiane’s ethos and praxis of life-art, and the feminist epistemologies embodied in Rubiane’s ethos and life-art praxis, what I call here a poetics of existence, in dialogue with debates on feminist epistemology and social reproduction (Federici, 2019).

This article mobilizes a concise theoretical scaffold. First, I draw on the coloniality/decoloniality corpus to understand how race, gender and geopolitics shape subjectivities and artistic labour (Quijano, 2007; Maldonado-Torres, 2007; Mbembe, 2001, 2016). Second, we use intersectionality to account for the co-constitution of race, gender, class and maternity in migrant trajectories (Crenshaw, 1991; hooks, 2015; Lugones, 2010, 2014). Third, diaspora studies help us situate displacement as an unfinished process of attachment and re-attachment (Safran, 1991; Clifford, 1994; Brah, 2005; Hall, 2006). Finally, performance studies and the notion of the open work ground our reading of Maia’s practice as an open score enacted with audiences and contexts (Schechner, 2017; Taylor, 2025; Eco, 1971).

## 2 Subjectivity(ies) in transit: migration, bodies, and arts-performativity

*Writing is evoking voices, listening to the bones, extending the body onto the page*. (Maia, 2023b, n.p.)

Before detailing the three dimensions that subtend this section, we outline the conceptual framework that guided our engagement with Rubiane Maia’s artistic work and trajectory. The potency of power structures in shaping our experience of the world is central to the constitution of personal histories. This becomes particularly evident across knowledge institutions and cultural fields. Yet how is this field formed? To what extent is subjectivity a space of freedom, and to what extent is it conditioned by social, historical, and cultural factors? The potency of power structures in shaping our experience of the world is central to the constitution of personal histories, and this becomes particularly evident within galleries and museums (Ellis et al., 2011; Spivak, 2021). These institutions, often marked by colonial-capitalist logics, configure spaces where dynamics of exclusion and oppression are reproduced, conditioning the subjectivities that inhabit them.

A decolonial approach to migration requires attention to epistemological inequalities between the Global North and Global South (Guerra, 2023b, 2023d). In our analysis of Maia’s trajectory, we draw on hooks (2015) and Lugones (2010, 2014) to frame how intersectionality and decoloniality amplify marginalized voices and unsettle dominant assumptions about migration. Such an approach challenges dominant narratives and fosters a more honest understanding of migratory experiences. It seeks to dismantle knowledge hierarchies shaped by colonial pasts, recognizing that migration has too often been studied through perspectives that disregard historical and social inequalities. Employing this approach demands that researchers not only acknowledge, but also integrate, the voices and experiences of migrants—particularly those from African and Latin American backgrounds who confront systemic racism and discrimination (Wassmansdorf, 2016).

Research rooted in decolonial perspectives is essential, as it reveals how colonial power structures continue to affect migrants’ lives. This, in turn, allows for the development of more just and inclusive policies attentive to lived realities. Intersectionality, viewed through a decolonial lens, is a crucial tool for developing a broader and more comprehensive understanding of contemporary migration dynamics (Crenshaw, 1991; Guerra, 2025b). Intersectionality operates through the recognition of multiple identities—race, gender, class, and sexual orientation, among others—that intersect and shape migratory experiences. It makes visible the simultaneous ways in which different forms of oppression manifest (Dogramaci and Mersmann, 2019; Mignolo, 2011). Applied to Maia’s trajectory (hooks, 2015; Lugones, 2010, 2014), intersectionality and decoloniality amplify the voices of marginalized groups, including women, LGBTQI+ people, and migrants, particularly those from racialized communities. It also challenges dominant assumptions about migration, which are frequently based on the experiences of white, heteronormative migrants. In this way, intersectionality enriches analysis by acknowledging the multiple dimensions of identity and the interplay of diverse forms of oppression, enabling a more holistic understanding of migrants’ lives and the social structures that shape them.

We read Maia’s migrant subjectivity through a theoretical lens on coloniality and racial capitalism (Mbembe, 2001, 2016; Maldonado-Torres, 2007; Quijano, 2007), understanding how historical, social, cultural and political forces shape performances that foreground the body (Mbembe, 2001, 2016; Maldonado-Torres, 2007; Quijano, 2007). These stand in opposition to dominant colonial subjectivities. Her performances, by articulating narratives of belonging and resistance, interrogate such structures and propose new pathways for subjectivity in the artistic field. In analyzing female subjectivity and migration in the arts (Guerra, 2021a, 2023c), it becomes essential to focus on the strategies employed by women to confront oppression, while equally making their life narratives visible. In this sense, their stories disrupt layers of the dominant logic, which is marked by colonial-capitalist unconsciousness and by anthropo-phallo-ego-logo-heterocentric norms (Rolnik, 2018; Sousa, 2024). Maia’s artistic practice is marked by the intersection of body, memory, and cultural-social territories, exploring themes of identity, migration, femininity, and resistance:

*I felt the impact of migration, even though I was coming within a context that was, in some sense, personal and loving, with a certain comfort, with the desire for a new life—not only professionally, but also personally. It was a difficult process with a profound impact on my life, especially because I migrated and became a mother at the same time, which meant a great change. First, I think it was an emotional impact. Migration placed me in a new country where I did not speak English, and that was very difficult for me. My relationship with the language was complicated because it deprived me of the autonomy to pursue the kind of work I had been doing. Even though it was visual, even though it was body-based, even though it was performance, it also involved communication with others, which was very effective and integral to the composition of my research in performance, in life, in encounters. I felt this loss of autonomy very strongly. I think that, in the experience of leaving a country like Brazil and living in Europe, there is this shadow of rejection—part of a system that refuses you before saying yes, and forces you to prove your right to be here. This had a profound impact on me. Despite being married, I had to marry legally in order to remain here. (Rubiane Maia, interview, April 2024)*

As Rolnik (2018) argues, subjectivity is not a fixed or static entity, but rather is constituted dynamically through agents’ relations with the environment, with the Other, and with structures of power. When Maia describes her simultaneous experiences of migrating and becoming a mother, a dual process of transition is revealed, one that reconfigures her position in the world. As Kloß (2025) notes, such a movement entails a reevaluation of modes of existence, as life is confronted with new linguistic, cultural, and affective codes. For Rolnik (2018), migration involves traversing not only physical but also symbolic territories, bringing about profound transformations in desires, plans, and ways of relating to oneself and others. This turbulence may destabilize psychic structures and open pathways to new lines of flight, while also producing mechanisms of exclusion and prejudice (Mahler and Pessar, 2006).

Maia repeatedly underscores her loss of autonomy due to the language barrier, particularly regarding her artistic and professional practice in England. Here, we can apply Rolnik’s (2018) notion of cafetinagem—the existence of social and political dispositifs that constrain subjectivity, undermining freedom of expression and creative potential. For Maia, as a woman, mother, and migrant, the language barrier became an instrument of control, inhibiting her performativity. If, for Rolnik (2018), desire is the motor of creation that traverses body, mind, and relationships, then the communicative limitation imposed by language functions as a form of imprisonment of that force. This imprisonment is further intensified by a migratory system that, as Maia notes, “refuses before saying yes.”

This is corroborated by numerous authors who have contributed to discussions on migration, decoloniality, intersectionality, and the experiences of marginalized groups, particularly within the contexts of migration policies and the realities faced by migrants and refugees (Ee Ling, 2023; Imray Papineau, 2024; Kalemba, 2021; Lacroix and Fiddian-Qasmiyeh, 2013). Their contributions are fundamental for advancing decolonial thought, promoting critical perspectives capable of challenging Eurocentric narratives, and valorizing subaltern knowledges and experiences.

Bhabha (2002) also offers a significant reading by introducing the concept of the “third space,” which conceives of culture as something dynamic and negotiated within contexts of cultural hybridity. In Maia’s case, the foreign language becomes a challenge that amplifies tensions between belonging and exclusion. It is in this “in-between”—the third space—that the sense of being refused “before even saying yes” is located, as she described in her interview. For Bhabha (2002), the migratory experience may give rise to new forms of subjectivity; however, when linguistic mastery is absent, the desired mobility (Gilroy, 1994) risks being captured by migratory control dispositifs that impose rules and barriers. Similarly, Brah (2005) discusses how diaspora spaces emerge from complex entanglements shaped by the intersections of race, gender, class, and nationality (Clifford, 1994). In Maia’s narrative, being a woman, mother, and migrant constitutes a triple intersection that intensifies vulnerability in the face of migration regimes. The language barrier thus functions as a mechanism that not only hinders social interaction but also renders the artist invisible and subalternized, limiting her access to professional networks and alternative cultural circles. Her performances and artistic creations, as we shall see, emerge as acts of resistance to these mechanisms of exclusion and control. In line with Kilomba (2020), who examines how everyday racism silences nonwhite voices (Ahmed, 2020), we can extend this reflection to xenophobia and linguistic exclusion. While not the same marker as race, the underlying logic is similar: systems elevate dynamics of rejection and silencing that compel constant proof of the right to exist in a given space. This process proves particularly violent when it affects the performative practice of an artist.

## 3 The body as a site of memory and resistance: elements of a biographical rupture

*There is a voice that says, shut up. shut up, all of you who are standing at the edge of the abyss*. (Maia, 2023b, n.p.)

Our approach to Rubiane’s work requires us to understand her artistic practice as a dialogue between body, landscape, and memory. In her performances, the body functions simultaneously as a repository of personal and historical narratives, embodying the tensions of migration, displacement, and cultural belonging, while rendering visible the often-silenced struggles of marginalized identities within the global art scene. A central aspect of Maia’s work is the concept of biographical rupture—a term she uses to describe the transformation that migration imposed on her identity and creative process:

*Before moving to England, I first began to travel. I made several international trips to participate in artistic residencies or projects abroad. I come from a world of performance, where encounter and presence strongly shape the possibilities of work. Initially, I started joining several projects and independent performance festivals around the world, through applications and invitations. I worked as a teacher, but used the holiday periods to engage in these enriching travel experiences, which brought me into contact with the works of many artists worldwide. This was also a process that had an impact on and enriched my own practice … Then I arrived in the UK and had to reinvent myself. I went through this process of artistic anonymity, for example. I call that moment a biographical rupture: I think I experienced a biographical rupture. (Rubiane Maia, interview, April 2024, our emphasis)*

**Figure 1:**
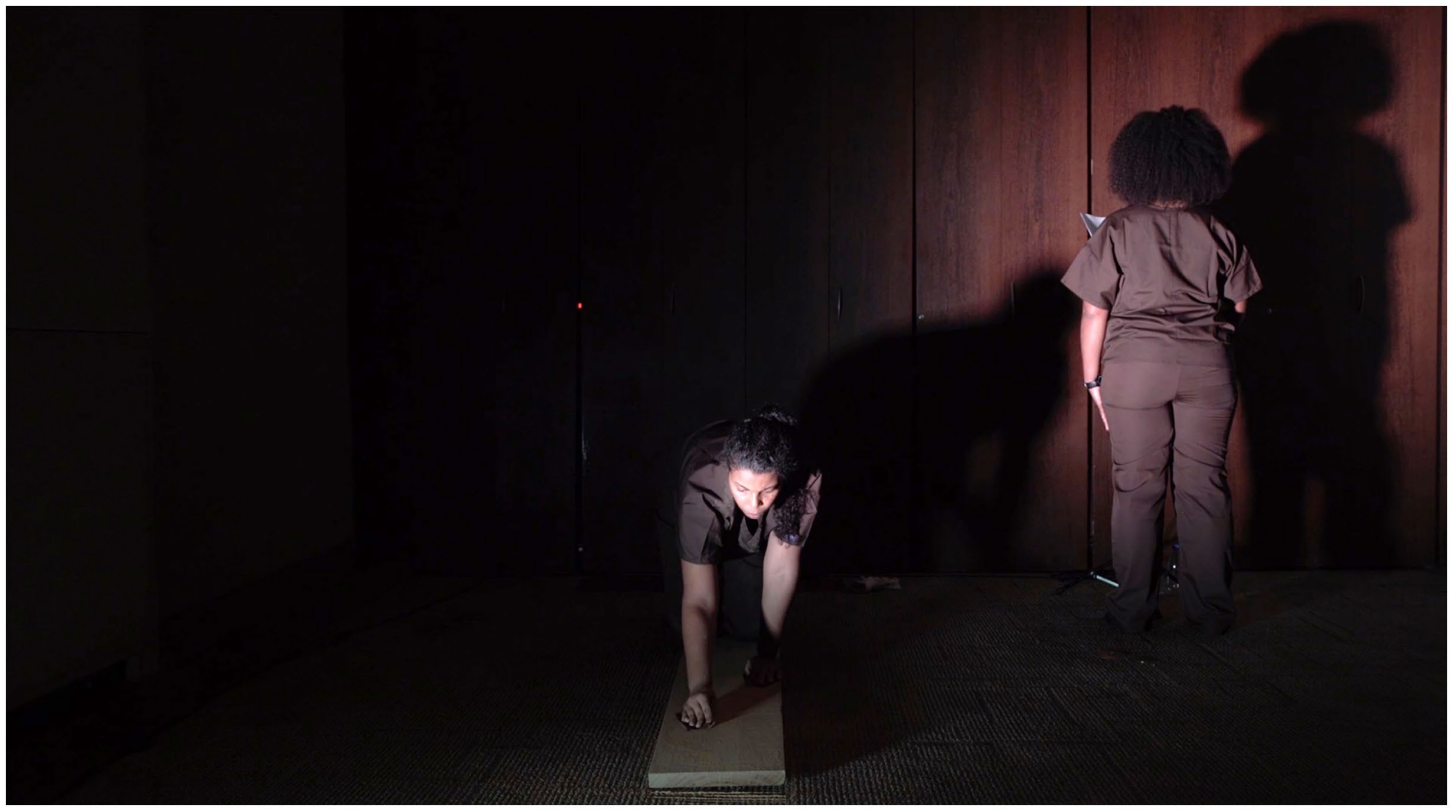
Rubiane Maia, *There is a voice that says, shut up. Shut up, all of you who are standing at the edge os the abyss I. Book-Performance, Chapter II*, 2018. Flexões Performáticas. Centro Cultural Banco do Brasil, São Paulo, Brazil. Images from video documentation. Courtesy of the Artist.

**Figure 2:**
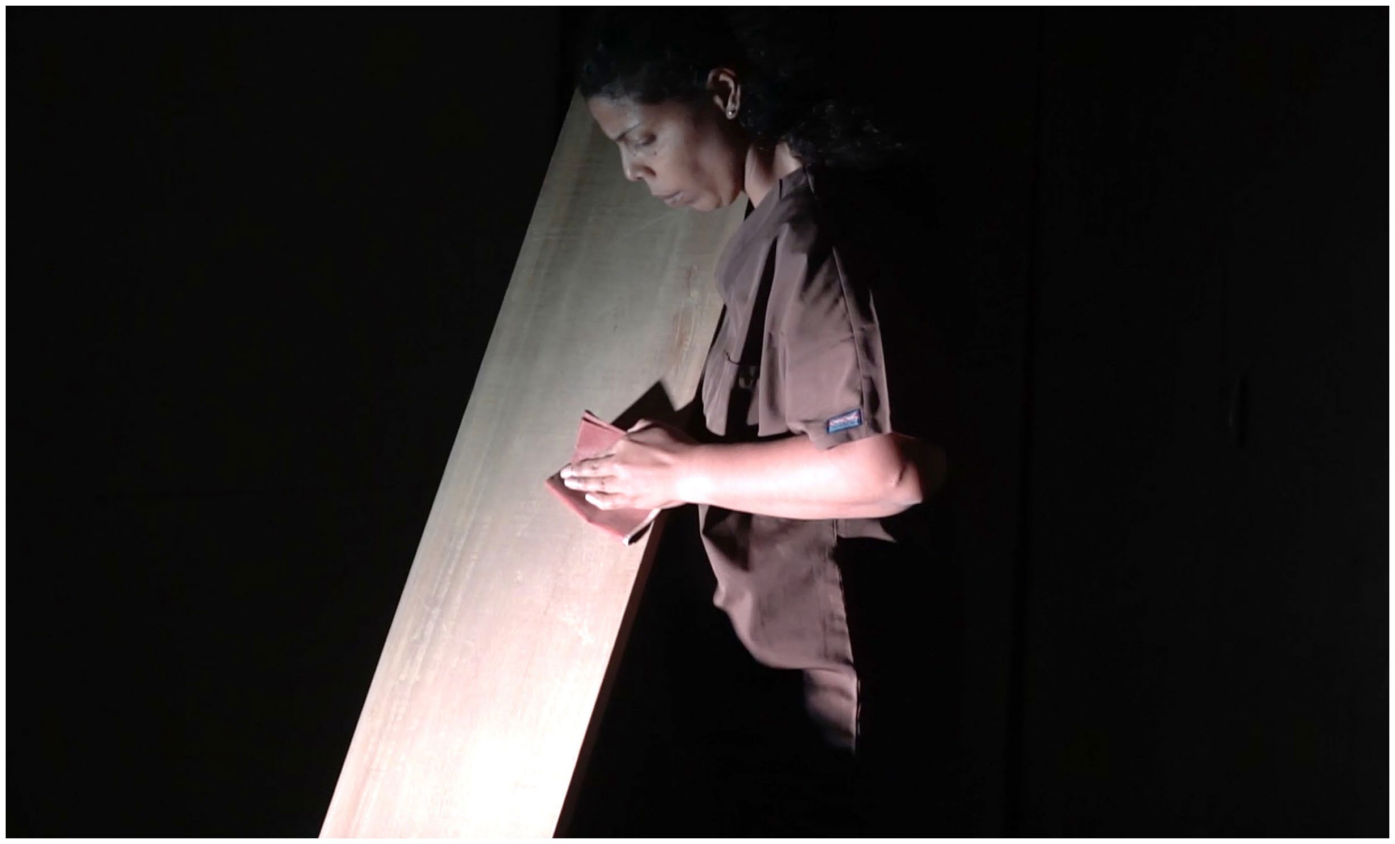
Rubiane Maia, *There is a voice that says, shut up. Shut up, all of you who are standing at the edge os the abyss II. Book-Performance, Chapter II*, 2018. Flexões Performáticas. Centro Cultural Banco do Brasil, São Paulo, Brazil. Images from video documentation. Courtesy of the Artist.

**Figure 3:**
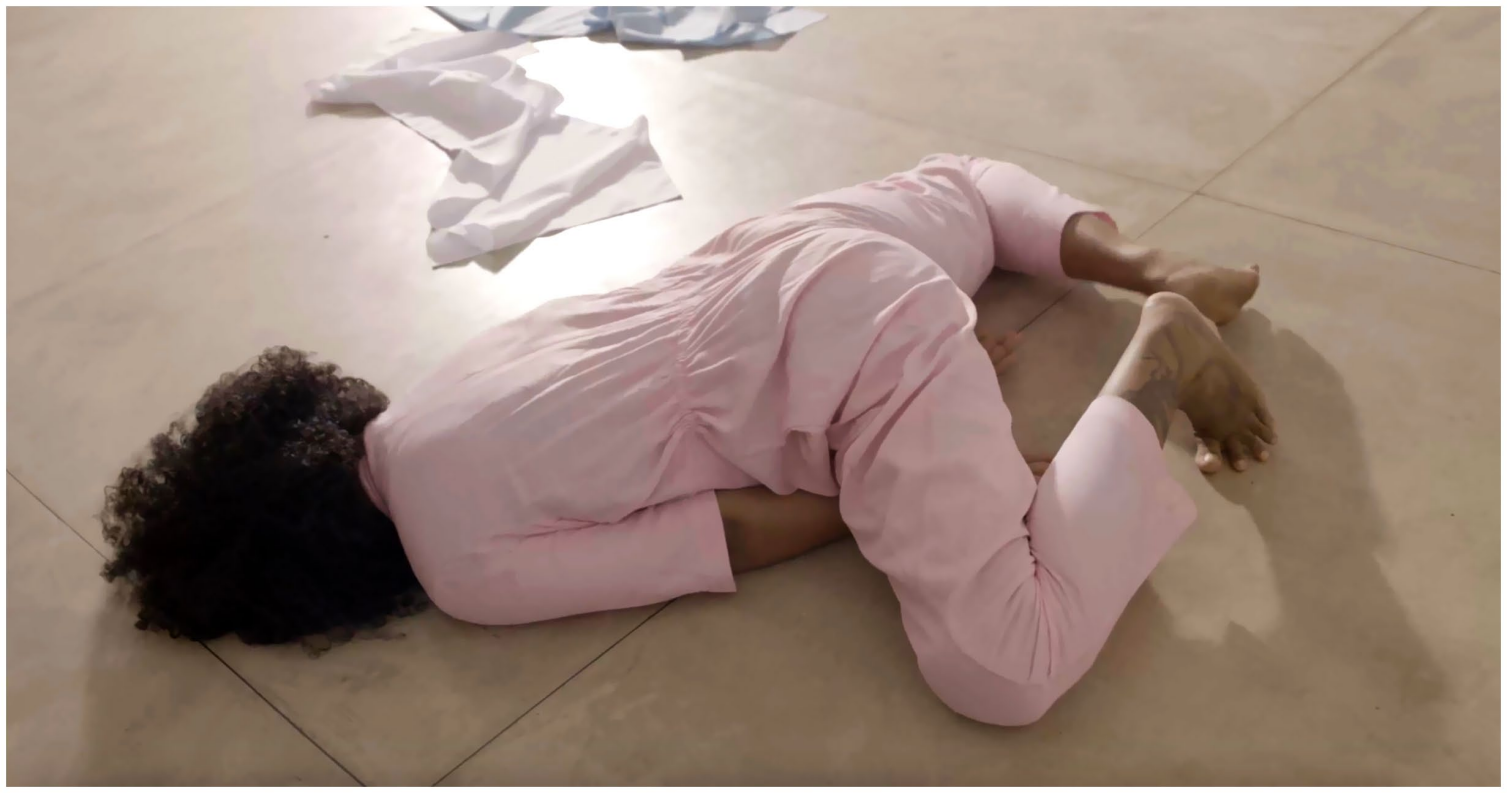
Rubiane Maia, *Just a slight relaxation of the mooring is enough for the boat to start dancing on the wave I. Book-Performance, Chapter IV*, 2020. Galeria Matias Brotas, Vitória, Brazil. Images from video documentation. Courtesy of the Artist.

**Figure 4:**
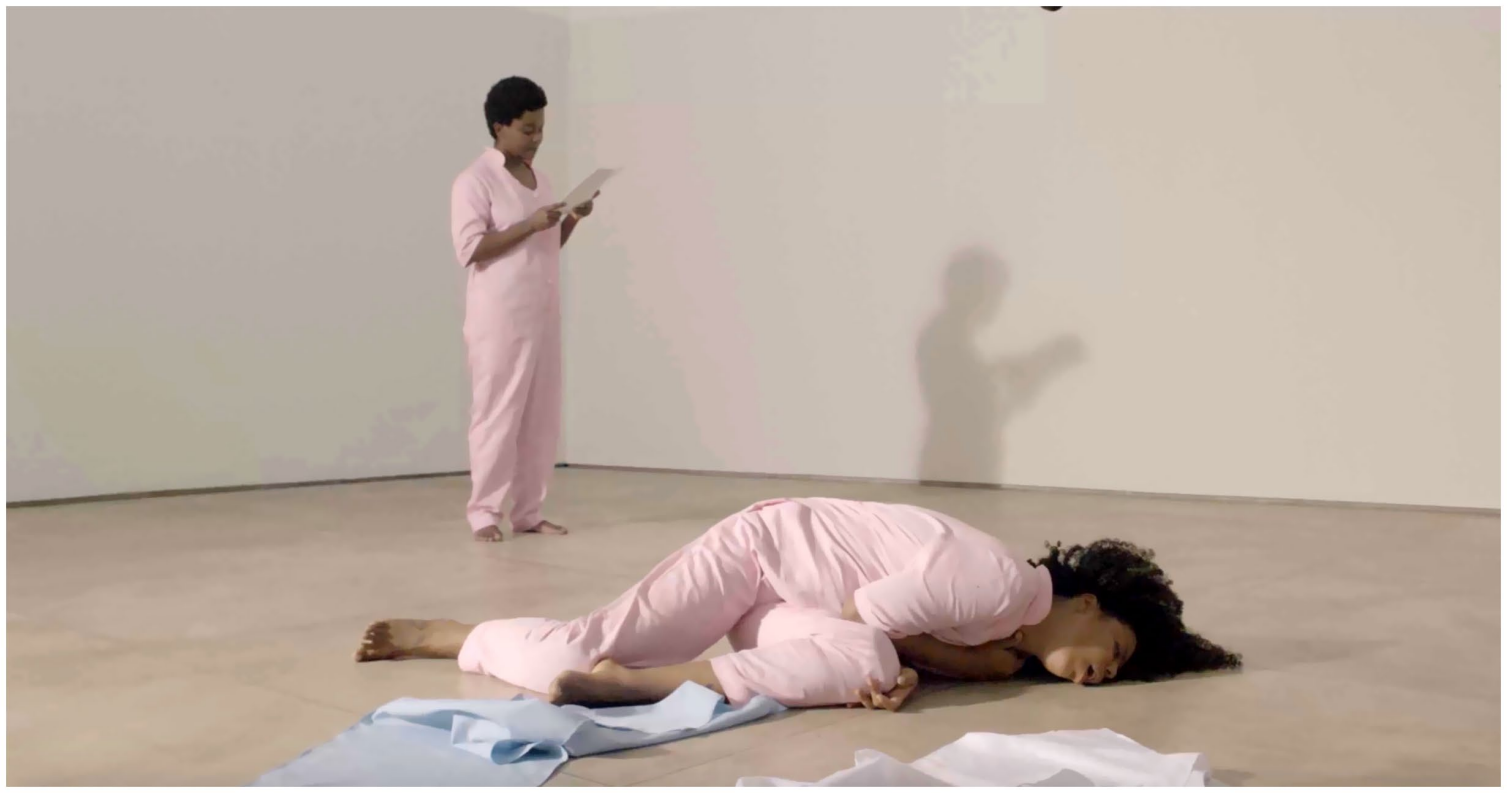
Rubiane Maia, *Just a slight relaxation of the mooring is enough for the boat to start dancing on the wave II. Book-Performance, Chapter IV*, 2020. Galeria Matias Brotas, Vitória, Brazil. Images from video documentation. Courtesy of the Artist.

Moving to England was not merely a geographical shift but a profound reconfiguration of the self—something Maia explores in her *Book-Performance*^5^ (see Figures 1–4). Through automatic writing and performative action, she investigates trauma, memory, and transgenerational pain, weaving an artistic language that interlaces personal and collective histories:

*I decided to write about the challenge of living in another country. My struggle with another land, another language, other bodies, and another atmosphere. I also decided to write about my difficulties with postpartum exhaustion and emotional collapse. At the time, I committed myself to writing something every day for a year, using automatic writing exercises and the psychographic practice proposed by the spiritualist Alan Kardec for communicating with the dead. To my surprise, these daily activities opened a channel for deep catharsis, in which layers of trauma related to motherhood, racism, and misogyny resurfaced from oblivion. After months, I realized the obvious: becoming both an immigrant and a mother at the same time provoked in me an abrupt biographical rupture. As a way of finding my voice, ancestral heritage, and inner strength, I slowly began to use language to (re)imagine, reconstruct, and rewrite fragments of my life story, incorporating the urgency of offering attention, listening, and care to the transgenerational traumas that affect the majority of Black people. Methodologically, the* Book-Performance *project became a series of related actions and texts, shaped according to the contexts of each new presentation. Yet all the pieces focus on one part of the body, with the aim of metabolizing through it complex or indigestible memories into small doses of healing and freedom. The* Book-Performance *is always presented by myself together with an invited collaborator—another Black woman*. (Maia, 2023a, n.p.)

The migratory experience is rarely neutral. In Rubiane’s narrative, displacement implies various forms of structural violence, loss, and existential rupture. Safran (1991) describes diasporas as communities marked by an originary wound—a founding trauma that shapes diasporic identity and, in Maia’s case, is refracted through her artistic creations. Clifford (1994) emphasizes the unfinished character of these trajectories, since dispersion does not end with departure but rather persists through affective, economic, and political ties that keep the wound of uprooting open. Biography is not a linear continuity, but a construction subject to breaks and continuities. Rubiane’s migratory trajectory reconfigured—and continues to reconfigure—identities and memories (Halbwachs, 1992): in *Speirein*, the artist reinscribes on the body the marks of displacement, transforming the trauma of separation and loss into an aesthetic and political gesture. By mobilizing the body as a space of memory inscription, Maia makes the biographical rupture not only a record of discontinuity but also a possibility of resistance and reinvention (Alexander, 2004; Lepecki, 2006; Pollock, 2003).

The discussion of traumatic migration converges around three complementary axes. The conceptualization of migratory trauma surpasses the classical notion of an isolated event. Since the late twentieth century, authors such as Safran (1991) and Clifford (1994) have emphasized that exile entails an originary wound for diasporic communities—a hiatus that remains open through affective, political, and economic ties. Alexander (2004) deepened this reading with the notion of cultural trauma, explaining how experiences of violence, persecution, or catastrophe reconfigure collective narratives of belonging, as Back (2016) also notes. More recent studies (Ee Ling, 2023; Kalemba, 2021; Theisen-Womersley, 2021) synthesize these contributions into the so-called “triple trauma paradigm”: trauma is experienced through violence or deprivation in the country of origin; through threats and violations during transit; and through post-settlement stress, in which xenophobia, legal precarity, and housing insecurity keep the initial wound alive (Theisen-Womersley, 2021). Despite these advances, significant gaps remain—particularly the scarcity of longitudinal intergenerational studies that follow families across multiple migratory cycles to assess the evolution of trauma and long-term resilience factors (Fassin and Pandolfi, 2010). Rubiane seems to address this gap by involving her son Tian and her partner in her artistic practice.

Her reflections point us toward thinking about subjectivity and artistic creation in displacement contexts, as illustrated by the preceding excerpt—where she reveals both the genesis and methodology of her writing-performance practice. The “decision to write” about migration and the “emotional collapse” of the postpartum period can be read as a form of autoethnography (Ellis et al., 2011), through which the artist-performer uses her own lived experience as a site of inquiry. The combination of automatic writing and psychography, inspired by Kardec’s spiritualism (Pereira, 2021), suggests a channel of communication with non-rational dimensions that may be understood as a kind of personal and collective unconscious. Echoing Taylor’s (2025) contributions, Rubiane refers to repertoire as what emerges in performance—bringing forth memories, embodied knowledges, and sociohistorical marks. In this sense, her daily writing practice constitutes an “intimate performance” that mobilizes dormant forces and traumas associated with misogyny, racism, and the ruptures produced by motherhood and displacement—by the condition of being “in-between”.

Hall (2006), discussing cultural identities, stresses that diaspora and displacement can generate processes of identity reconfiguration, which are often traversed by anxieties and reinventions, as observed in other contexts (Guerra, 2020b, 2021a, 2023d). In Rubiane’s case, this process activates transgenerational narratives and memories (“affecting the majority of Black people,” as she noted in the interview), resonating with Hartman (2025) reflections on how colonial history and structural racism reverberate in the present, producing both bodily and psychic marks (Ahmed, 2020). Her mention of “transgenerational traumas” underscores the importance of confronting collective wounds historically transmitted through racial, gendered, and colonial violence. Collins (2000) points out that Black women’s experience is marked by multiple oppressions, but also by a collective resilience expressed through practices such as speech (Ribeiro, 2017), writing, and performance. The catharsis provided by Maia’s daily writing becomes a form of care of the self (Foucault, 1997).

Turning to the *Book-Performance*, Rubiane notes that each piece focuses on “a part of the body” in order to “metabolize complex or indigestible memories.” In line with Foucault (1979), Maia suggests that the body is traversed by power relations that leave marks, which can in turn be reappropriated in practices of freedom (Fassin and Pandolfi, 2010). Moreover, as a feminist performance, this work reveals the body as an archive capable of reenacting and resignifying past violences, converting them into poetic acts of resistance and affirmation (Schneider, 2013).

Rubiane Maia’s *Book-Performance* is a hybrid artistic practice in which text, body, and memory intertwine to create a performative outcome. Drawing on different media (book, voice, body, presence, collaboration with other invited artists), Maia proposes an embodied form of writing that transcends conventional reading and invests in the relational and sensorial dimension of performance (Schechner, 2017). From this perspective, each piece becomes a kind of ritual that combines the materiality of the written word (inscribed in pages, notebooks, or projections) with the physicality of bodies in action. To better grasp this artistic proposal, we return to Taylor’s (2025) distinction between archive (documents, books, records) and repertoire (enactments, gestures, performances). In this work, Maia seems to fuse both, since the book is not a static support but a platform for corporeal and vocal action—reactivating memories and narratives (Kilomba, 2020). The potency of the work lies in this hybridity (Harrison et al., 2018), specifically in the written text that is simultaneously enacted, (re)read, and transformed into an ephemeral event—into a performance that reconfigures and renews the meaning of words in each presentation (Eco, 1971).

Another key point in our analysis is how Rubiane engages with corporeality, particularly in relation to Black female identity (Davis, 2011; hooks, 1995). Butler (1997b), in discussing performativity, argues that identities are not fixed but rather constructed through repetitions and acts that may reproduce or subvert norms (Butler, 2013). In the *Book-Performance*, subversion occurs as the body (Black, maternal, immigrant) takes center stage, claiming the right to narrate its own story and to embody memories that have historically been silenced. At the same time, the notion of writing of the self (Foucault, 1997) acquires a collective dimension, since this performance not only expresses Maia’s subjectivity but intertwines it with that of other women, producing symbolic cartographies (Sotelo-Castro, 2010) wherein experiences of motherhood, migration, and ancestry are updated and transformed into poetic actions. Thus, Maia’s work can be read alongside the “diasporic cartographies” analyzed by Brah (2005), in which the experience of displacement—geographical and/or symbolic—finds meaning in sharing and (re)creation. In an interview, Rubiane explains:

*This* Book-Performance *project began in 2018. And before it became a project, it began as an experience with writing—finding in writing both elaboration and catharsis. I think before elaboration came catharsis, because I started using a method of automatic writing that related to psychographic experiences. I did this at a moment when I needed to anchor myself, because it was exactly the transition moment I’ve already mentioned—coming here—and it was during my postpartum period, when I was experiencing this vulnerability, this biographical rupture. In the midst of everything, the only thing I somehow felt capable of doing was writing. I started writing every day. And I did this as a kind of personal commitment. I wasn’t thinking of it as an artistic project, but simply as a commitment: I will sit down and write. And I won’t write a story, because my goal wasn’t for the writings to become anything. I would just write whatever came into my mind. As I sat down to write, many things started to surface, including uncomfortable ones. I realized it was very difficult to write about discomfort. And this deepened to the point where I wondered: how do I switch off, how do I remove this filter, how do I deactivate—yes, that’s the word—this filter that tells me, don’t write this, don’t tell this, don’t think about this. I began doing exactly the opposite: trying to write without reflection, just writing. And it became a thread I kept pulling, and a lot came out. I started doing this every day. It was an intense period, because through this process I began glimpsing my relationship with traumatic processes—not only mine, from my own life and experiences, but also transgenerational ones. Stories I had always believed happened a certain way began to resurface differently. They came not only through the narratives I had always heard but in other forms. I allowed those voices to gain strength, and it turned into a very entangled, cathartic writing process, a constant rewriting, a writing about writing itself, with many overlapping layers, often without beginning or end. (Rubiane Maia, interview, April 2024)*

Maia’s work *Speirein*, developed in 2019, also exemplifies this engagement with trauma and migration. Using fragmented sculptural elements of feet—symbolizing movement, journey, and rupture—she invokes the struggles and resilience of bodies in transit. Inspired by the transatlantic crossings of her ancestors and the contemporary crises of forced migration, *Speirein* operates as a poetic-political declaration, bringing to light the lived experiences of displacement and the scars it leaves. The word *Speirein*, derived from Greek, means to sow, to scatter, to disperse. Some scholars (Clifford, 1994; Safran, 1991) suggest that *Speirein* gave rise to the term “diaspora,” which also originates from classical Greek and denotes the dispersion or displacement—often forced—of populations from a determined territory to various destinations. In general terms, it means the dispersal of a nation or ethnic group throughout the world. From a sociological perspective, diaspora refers to the dispersion of ethnic or national groups that, for diverse reasons, relocate from their homelands to other host spaces. This concept has gained increasing relevance in debates on contemporary migration, particularly in light of refugee crises and displacements that expose the complex web of global vulnerabilities (Rolnik, 2018). By naming her work *Speirein*, Maia updates this conceptual tradition, anchoring it not only in the memory of her ancestors’ transatlantic crossings but also in the problematization of present-day forced migrations.

**Figure 5:**
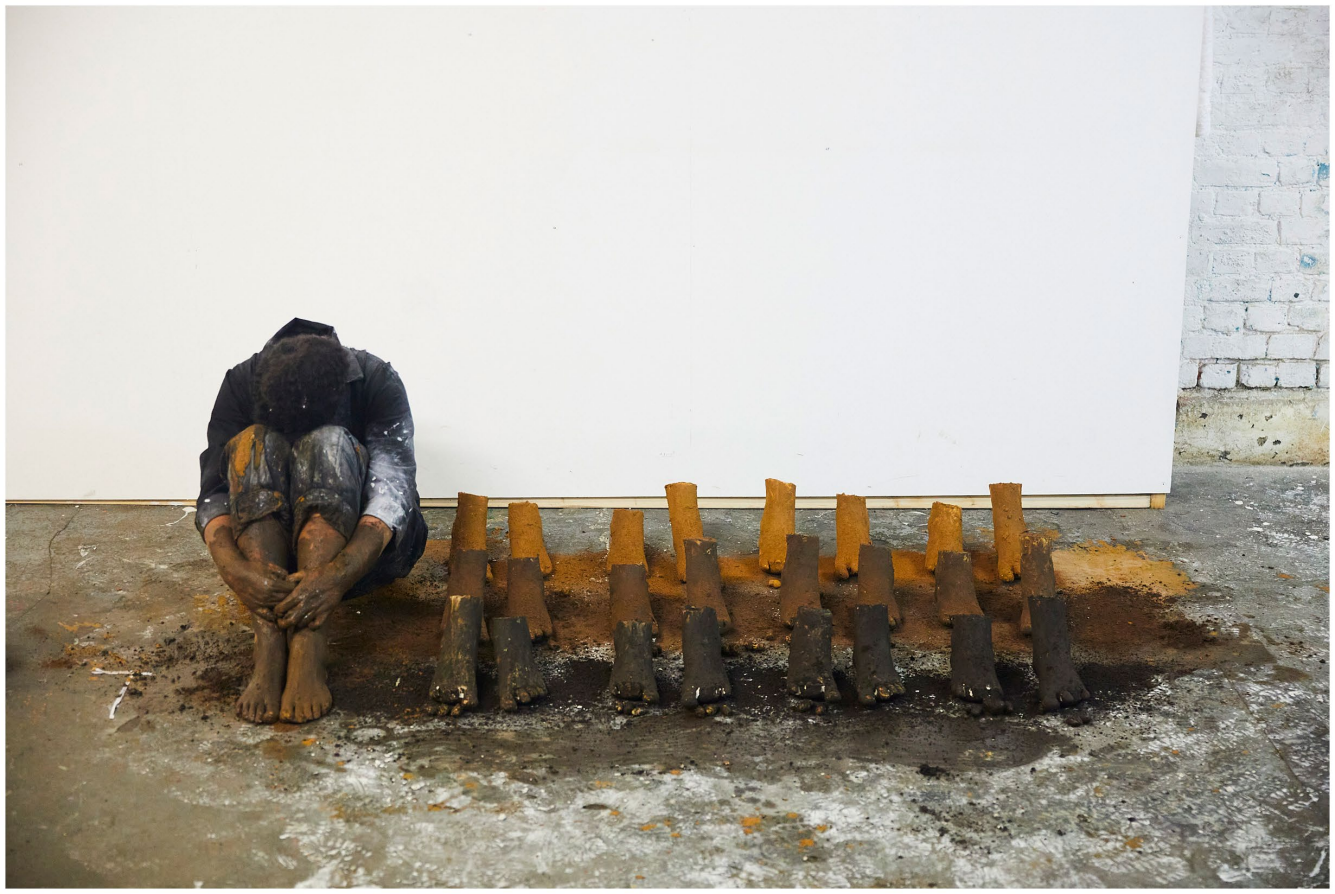
Rubiane Maia, Speirein, 2021, 10-hour performance I. PSX: a decade of performance art in the UK, The Ugly Duck, London, UK. Pictures by Manuel Vason. Courtesy of the Artist.

**Figure 6:**
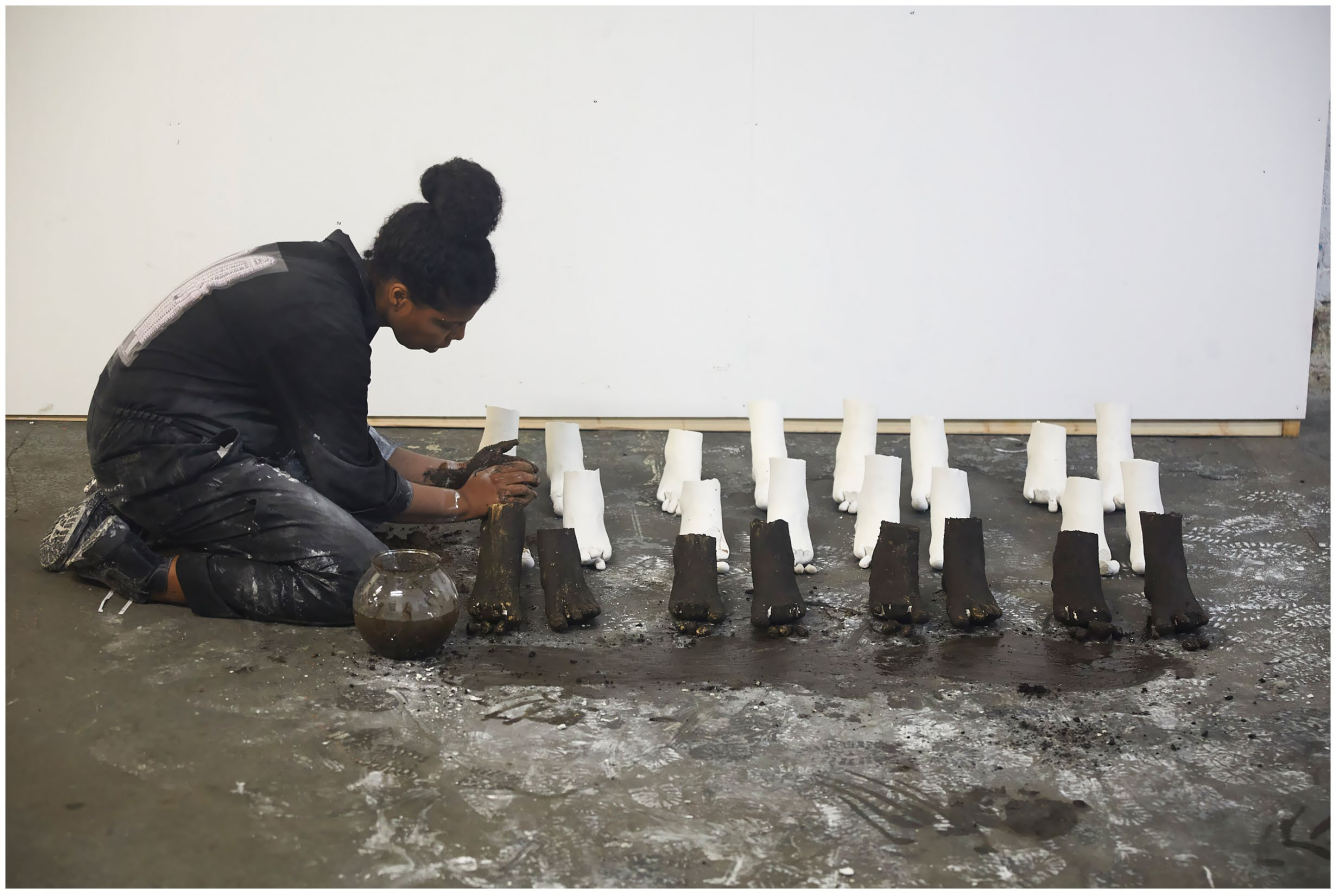
Rubiane Maia, Speirein, 2021, 10-hour performance II. PSX: a decade of performance art in the UK, The Ugly Duck, London, UK. Pictures by Manuel Vason. Courtesy of the Artist.

**Figure 7:**
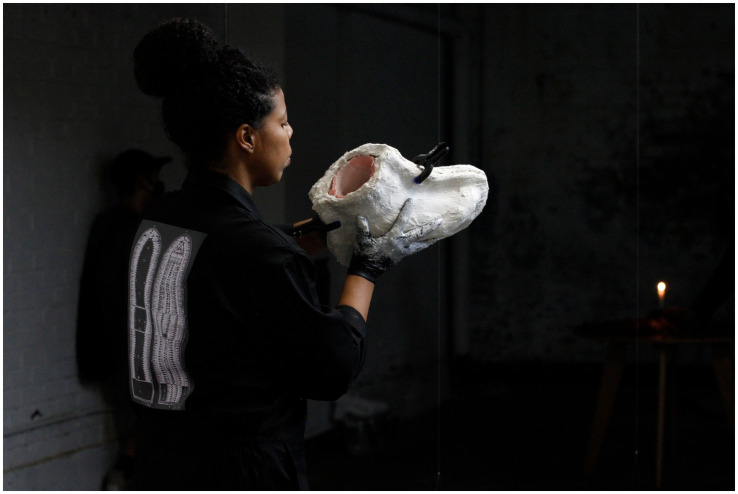
Rubiane Maia, Speirein, 2021, 10-hour performance III. PSX: a decade of performance art in the UK, The Ugly Duck, London, UK. Pictures by Manuel Vason. Courtesy of the Artist.

Rubiane Maia situates herself within a lineage of creation and reflection that transforms lived experience into critical discourse, aligning with perspectives such as those of Rolnik (2018), Lugones (1992), and Kilomba (2020), who highlight the importance of reactivating historical-traumatic memories through artistic practices that summon bodies and subjectivities into the exercise of reexistence. In *Speirein*, Maia worked continuously for 10 h, producing multiple molds of her own feet. This long-duration, repetitive action was divided into four stages: in the first, she prepared the raw material—a mixture of plaster, cement, water, and a fast-drying powder. In the second, under the guidance of a mold and sculpture specialist, she poured the mixture into silicone matrices. In the third step, she kept the matrix stable until it dried completely. Finally, in the fourth step, she removed the hardened piece from the mold. The continuous process lasted a total of 9 h, culminating in three rows of 12 pairs of feet placed on the ground. Many bore interesting marks, such as amputated toes or fractures incurred during removal. In the final hour of performance, Maia introduced brown and black soil (collected from tree roots) and rainwater in a ritualistic act: massaging each white plaster foot with the damp earth, staining them, and finally painting her own feet (Figures 5–7).

In the last stage of the performance, the artist reintroduces ritualistic and agrarian dimensions (*Speirein* = to sow), using different soils and rainwater to transform the white plaster surface into something hybrid, layered with memory. This repetitive act of “planting” and “spreading across the floor” invokes an ancestral connection to land and displacement. The white plaster—conventionally associated with purity or neutrality—becomes something hybrid, charged with memory and tied to the Black Atlantic (Gilroy, 1994), where cultures and bodies circulate in the context of African diaspora. In *Speirein*, earth becomes an element reconnecting people to origins even in contexts of removal, carrying the dual meaning of exile and rebirth (Guerra, 2021b, 2023c, 2023d).

From a sociological perspective, *Speirein* resonates with reflections on diaspora as an ongoing process of identity reconstitution (Brah, 2005; Hall, 2006). The sculpted feet emerge as metaphors of interrupted, forced, or unfinished journeys. The repetition, duration, and persistent fractures reiterate the traumatic dimension of collective displacements. This is a memory of/in the body (Guerra, 2025a, 2025b), one that traverses generations and geographies and that Maia’s art reactivates, giving visibility to narratives of resistance (Sousa, 2024). For contemporary understandings of forced migration, *Speirein* offers not only a poetic expression of trauma (Theisen-Womersley, 2021), but also a political declaration that underscores the tensions of a globalized world—rigid borders, xenophobia, struggles for recognition, and the citizenship claims of those in transit (Lepecki, 2006; Pollock, 2003). Art in general—and *Speirein* in particular—becomes a space of mediation between macrosociological discourses and the singular experiences of each displaced subject (Back, 2016).

## 4 Decolonial and intersectional feminist epistemologies and the complexification of artistic creation

*This voice cuts me off, removing my feet from their place*. (Maia, 2023b, n.p.)

Maia’s art-life engages viscerally with contemporary feminist and postcolonial theories, particularly those addressing the intersections between gender, race, and geopolitics. Although Rubiane’s initial academic experiences did not include a critical engagement with feminism and racial issues, she later found in Black feminist thought a fundamental framework for her artistic inquiries (Klein and Toledo Ferreira, 2024). Inspired by authors such as hooks (1995), Kilomba (2020), and Gonzalez (1979), Maia critically examines the role of Black women in art institutions and the mechanisms that perpetuate their invisibility:

*I think that, unfortunately, the colonial legacy is within us. And we have to deconstruct it all the time. We have to build our sense of relation to what surrounds us; and we have to deal with our desire to control and to exploit. I’ve been thinking that every time I go somewhere to perform, for me the encounter with that place is a work in co-creation with that environment. And so, no matter how much I have a project, a desire to construct an image, a specific action—that will only happen if the environment is favorable, if the environment also says yes to it. I think that in my work, I have tried to open myself up to a more complex way of thinking in relation to urgencies. I don’t have a specifically racial agenda, I don’t have an agenda only directed at climate and ecological issues, but I have been thinking a lot about processes of alterity and how we transform ourselves collectively, how we take responsibility for processes of transformation.*

Her performative practice is situated within a context of decolonial and intersectional feminist resistance to biased processes of alterity, challenging the normative structures of the art world. Rubiane highlights the performative paradox within institutional spaces, where diversity is valued in discourse but rarely materialized in praxis. Her presence in predominantly white European art circuits reveals the persistence of racial and gender hierarchies, making her art an act of survival and affirmation—a dimension that has shaped her trajectory from the outset:

*I am the first in my family to have access to university: my parents never had the opportunity to study at university. They had to interrupt their studies early because of life circumstances. I come from a working-class family, who needed to work every day to support themselves. So our life was one conditioned by this process of putting work as the priority, and study came only insofar as you could manage it within economic life. Unlike the very limited opportunities my parents had, they always encouraged us to study. Study represented something important. So I studied, and I had the opportunity to go to university: I went to a public university. At the time I entered, quotas for Black people did not yet exist, so it was a very challenging process at the time, not something simple for me. (Rubiane Maia, interview, April 2024)*

This trajectory also unveils the systematic reiteration of feminist epistemologies. In this respect, the artist from Espírito Santo states:

*I think that, in general, feminist epistemologies came quite late to me, because, for example, at the time I began to study the arts in my undergraduate degree, I didn’t have training that was connected to critical thought about feminism and racial issues … Gender issues remained very much at the margins. These only began to appear when I started my master’s, and then I began to access a series of discussions and readings I had never had before. And it grew. But I think that, for me, feminism arrives at that moment of questioning in relation to racial issues—when feminism becomes a feminism that thinks about Black people, Black women … Today we talk about ecofeminism. We are creating a series of derivations of these questions which, in some way, intersect in this idea of rethinking what we are doing to ourselves in this world. And I think, for me, this is the central question, because it is impossible to talk about creating categories without, at the same time, touching on a fundamental issue: questions of justice, of greater equity, and how we navigate this world through processes of reconstitution and of living—of constructing different ways of life, of accepting difference. (Rubiane Maia, interview, April 2024)*

This testimony illustrates how feminist epistemologies—understood as bodies of knowledge that question and transform the patriarchal, colonial, and racist foundations of knowledge production (Guerra, 2025b)—arrived late in her formative trajectory. The artist points out that during her undergraduate years she did not access critical thought directed toward gender and race, but later began to read and engage with authors and debates encompassing intersectional feminisms, ecofeminism, and postcolonial discussions. This discovery resonated in her artistic practice, leading her to develop work attentive to a feminism that centers the specificities of Black women—an experience fundamental to her process of identification and artistic creation. In the artistic field, as well as in the training of artists, feminist epistemologies may be understood as critical positions that interrogate hegemonic patterns of knowledge and cultural production, demanding the inclusion of voices that are systematically silenced (Collins, 2000).

In Rubiane’s case, the belated perception of feminism and its ramifications (Black feminism, ecofeminism, decolonial feminism) reflects the critique that Black feminist authors have been making for decades: the absence of an intersectional perspective in institutional spaces of education. As hooks (2015) reminds us, many Black women only find feminism when it expands beyond a Eurocentric, elitist bias—when it recognizes race, class, and culture as dimensions of gender inequality. The artist under study relates this process to the fact that the feminism that makes sense to her is that which thinks about “Black people, Black women,” evidencing the primacy of feminist theories that operate in intersection with decolonial thought and anti-racist struggles. In this sense, the decolonial dimension—wherein the colonial legacy within power relations is criticized—finds resonance in authors such as Lugones (2020), who proposes the concept of the coloniality of gender to expose how the modern/colonial system instituted the subalternization of non-white bodies. Such a decolonial perspective connects to reflections on ecofeminism, as mentioned by Maia, insofar as both recognize racial, gender, and class oppressions in correlation with environmental exploitation (Mies and Shiva, 2014).

Another salient point in Maia’s testimony is her emphasis on justice, equity, and the recognition of difference as fundamental to rethinking life and modes of existence, whether through artistic creation or otherwise. By underlining that feminist knowledge cannot be produced without considering colonial legacies, racialization, and ecological dimensions, the artist aligns herself with Haraway’s (1995) formulations, which stress the importance of situated knowledge—that is, knowledge anchored in specific contexts, requiring recognition of the positionality of the knower (the researcher, the artist, the Black woman). In this sense, Rubiane’s artistic practice—shaped by her conditions and lived determinants—converges with what Haraway (1995) calls a politics of location, since it emerges from concrete lived experiences.

The broadening of feminism’s scope, as described by Rubiane, reflects the evolution of feminist epistemologies themselves over recent decades. If once feminism was more universalizing/universalized, today there exists a multiplicity of feminisms—Black, Indigenous, decolonial, ecological—each contributing to deepening the critique of systems of oppression and inspiring transformative artistic and political practices (Lugones, 2010). In Maia’s work, this expansion translates into performative investigations that address identity, affect, and corporeality, engaging directly with the concerns of a feminism that integrates intersectional, historical, and ecological dimensions. Her artistic production can thus be understood as a living expression of emergent feminist epistemologies, which cross disciplinary borders and forge new methodologies of creation and reflection (Imray Papineau, 2024).

Feminist epistemologies depart from the recognition that all knowledge is situated (Haraway, 1995)—that is, dependent on experiences, social positions, and embodied realities. This perspective entails a critique of hegemonic forms of knowledge that tend to render invisible voices at the margins of power structures (Choi and Greenhill Hannum, 2025). Accordingly, feminist epistemologies advocate for methodologies attentive to lived experience and the articulation of knowledges that address gender, race, class, and sexuality in an intersectional manner.

Elsewhere, Guerra (2023b, 2023c) mobilized the concept of minor theory as a set of discursive and existential practices emerging within a dominant language or system of power, but functioning as lines of flight (Deleuze and Guattari, 1975). This concept, derived from Deleuze and Guattari (1987), proves heuristically powerful for understanding Rubiane’s positioning and work. These authors argue that minor theory consists in the counter-normative use of a hegemonic language in order to produce deterritorializations and expose amplified social and political contradictions. Although enacted within the system, these minority expressions do not submit to it, but instead subvert it. Just as minor theory displaces and reconfigures the official language from the margins, feminist epistemologies likewise arise from subalternized positions in academia and society to demystify canonical scientific discourses. All of Rubiane’s research-creation inscribes itself here: for this artist, it is crucial to embrace flows, variations, differences, margins—legitimizing voices and bodies situated on the peripheries of official discourses (Crenshaw, 1991). In this sense, minority ceases to be synonymous with subordination and becomes a force of creation and resistance, an “in-between” of poetic possibilities.

## 5 Trajectories, bodies and borders … always *in between*. Final remarks

*I have used memory as an investigative device that involves remembering and reframing the numerous traumas and scars inherited by colonialism, which, generation after generation, are perpetuating themselves through naturalisation, silence and shame*. (Maia, 2023b, n.p.)

The trajectory and artistic practice of Rubiane Maia reiterate that the migratory experience is not merely a geographical displacement, but involves profound transformations in processes of identity construction and in the realm of power relations. Her artistic works, as analyzed here, foreground a set of issues traversing race, gender, class, and motherhood, highlighting tensions and contradictions lived in the process of migration and self-(re)invention. They reveal an in between trajectory of displacement, of searching for meaning, and of belonging.

This investigation of Rubiane’s journey, her circumstances, and her body of work has shown us that the body is a privileged locus for the inscription of traumas, resistances, and narratives, as underscored by decolonial and intersectional feminist epistemologies (Haraway, 1995; Lugones, 2014). The female, Black, and immigrant body experiences distinct dynamics of exclusion and vulnerability, while simultaneously becoming a vehicle of creation, subversion, and negotiation with the very structures that seek to control it. In Rubiane’s case, the performative body acts as a living archive of transgenerational memories and as a surface of resistance, insofar as the artist transforms her personal experience into a collective aesthetic and political practice.

Moreover, feminist epistemologies emerge decisively in understanding Rubiane’s trajectory. Even though they reached her somewhat belatedly, such perspectives—particularly those anchored in intersectionality, Black feminism, and the decoloniality of gender (Crenshaw, 1991; Gonzalez, 1979; Kilomba, 2020; Lugones, 2020)—provide the tools to analyze how racial, class, and gender dimensions interweave within her artistic and lived practice.

In this confluence, Maia’s artistic work acquires an expanded political meaning: it brings silenced colonial memories to the fore and contributes to plurality in the arts. Above all, we are speaking of displacements, of migration, of flows, of borders. And here lie all the questions that can and must help to complexify the approach to the contemporary artistic field, exposing inequality, injustice, and domination. Rubiane’s oeuvre and trajectory demonstrate that it is not merely about refusing the romantic epitome of “art for art’s sake,” nor the more recent postmodern epitome of “making life itself a work of art” (Lipovetsky and Serroy, 2014). Rather, it is about a holistic artistic project of life—where body and soul converge to claim a place in and through difference.

By examining the relationship between migration, art, and performativity, we see how migratory processes—even when traumatic—can be resignified through artistic creation. The concept of biographical rupture adopted by the artist proves decisive in revealing the intense subjective and structural changes triggered by the physical and/or symbolic deterritorialization that occurs when one moves to a different place from where one was born or departs from the country of origin. It is in these crossings that new “in-between” identity cartographies emerge, wherein performance and the body operate as dispositifs of reexistence, clearly assuming a position beyond the traditional hierarchical colonial positions of center and periphery/exterior (Anzaldúa, 1987).

Indeed, it seems that Rubiane mobilizes Antoszek’s (2022) concept of border *doxa*: the naturalized and internalized beliefs concerning the necessity of physical and symbolic borders. Borders, in this sense, are seen as something given, unquestionable, and necessary for the organization of space and life. What Rubiane proposes is a challenge to this doxa, interrogating the rigidity and function of borders—both physical and symbolic. She resists the reductive Aristotelian binarism that has tended to perpetuate structures, in favor of the liberating reticularity of flows that fosters and strengthens agency (Mignolo, 2011).

In sum, by articulating a decolonial–intersectional framework with performance studies, this article shows how Maia’s Book-Performance and Speirein convert migrant vulnerability into a form of aesthetic and political agency. Empirically, we contribute a case-based account of how Black, migrant, maternal subjectivities are negotiated through artistic labour; theoretically, we clarify how coloniality and the open work inform such negotiations. Limitations of this article include the single-case design and reliance on one semi-structured interview, thus, future research should compare multiple artists and follow intergenerational dynamics over time.

## Data Availability

The original contributions presented in the study are included in the article/supplementary material, further inquiries can be directed to the corresponding author.

## Footnotes

1 This formulation also echoes the 35th São Paulo Bienal theme, Choreographies of the Impossible (2023), in which Maia took part through the Vitória travelling program (see Maia, 2023a). This intertext clarifies how her practice situates choreography as a critical idiom for border experiences.

2 Rubiane Maia assumes herself as a visual artist, performer, writer and researcher, whose production transits between performance, installation, photography, video, writing and rituals (Maia, 2023b).

3 The artist has a spectrum of striking works that intersect performance, installation, photography, video, writing, and rituals. Her work is crossed by themes such as memory, body, language, Afro-diasporic spirituality, decolonization, and migration. Rubiane defines herself as a body that writes and performs as a method of investigation (Maia, 2023a), articulating artistic and autobiographical processes with situated critical thinking. Thus, among her works, we highlight *À Flor da Pele* [Flower Skin] (2011); *Travessia* [Crossing] (2012); *Decanto, até quando for preciso esquecer (Parte I)* [I am decanting, until I need to forget (Part I)] (2013); *O Jardim* [The Garden] (2015); *Preparação para Exercício Aéreo, o Deserto e a Montanha* [Preparation for Air Exercise, the Desert and the Mountain] (2016); *Livro-Performance* [Book-Performance] (since 2018); *Speirein* (2021); *Magical Curves* (2022); *Reimagining Care* (2023); *Contra-êxodo: estratégias de inserções* [Counter-exodus: strategies for integration] (2024); and *A conspiração dos ícones* [The conspiracy of icons] (2025).

4 By “resonating” I refer to Eco’s understanding of the artwork as an open, indeterminate structure that invites co-creation by interpreters. Thus, Book-Performance operates precisely as an open score—its texts and actions are re-configured in each presentation, fusing archive and repertoire.

5 Rubiane defines that the *Book-Performance* “is an ongoing project composed by a series of actions devised in response to autobiographical texts particularly influenced by traumatic transgenerational memories connected to gender and race issues” (Maia, 2023b, n.p.).
